# A Case of Acquired Pars Flaccida Cholesteatoma Extending to the Supralabyrinthine Petrous Apex With Preservation of the Otic Capsule

**DOI:** 10.1155/crot/2654191

**Published:** 2026-06-22

**Authors:** Hisashi Sugimoto, Manabu Inaba, Goki Nagasaki, Tomokazu Yoshizaki

**Affiliations:** ^1^ Department of Otolaryngology-Head and Neck Surgery, Kanazawa University Graduate School of Medical Science, Kanazawa, Japan, kanazawa-u.ac.jp

**Keywords:** middle cranial fossa approach, petrous bone cholesteatomas, supralabyrinthine

## Abstract

**Background:**

Supralabyrinthine petrous bone cholesteatoma is a rare subtype in which hearing preservation may be achievable depending on the anatomical extent and surgical approach. Appropriate selection of surgical strategy is essential.

**Case Presentation:**

A 31‐year‐old man presented with a 2‐year history of persistent left otorrhea. Otoscopic examination revealed pars flaccida cholesteatoma. Pure‐tone audiometry demonstrated hearing loss with an air–bone gap of 35 dB. Computed tomography showed soft‐tissue density extending from the tympanic cavity and mastoid air cells to the supralabyrinthine petrous apex. Inner ear function was almost preserved.

**Intervention:**

A staged surgical approach was adopted due to active inflammation. First, canal wall–down tympanomastoidectomy via a transmastoid approach was performed to control infection. Six months later, second‐stage surgery was performed using the middle cranial fossa approach for complete removal of the supralabyrinthine cholesteatoma, along with ossicular chain reconstruction.

**Outcome:**

No intraoperative or postoperative complications occurred. Postoperative non–echo‐planar diffusion‐weighted MRI at 4 months showed no residual disease. At 22 months, hearing improved with an air‐conduction threshold of 21.7 dB, and no tympanic membrane findings suggestive of recurrence were observed.

**Conclusion:**

In supralabyrinthine petrous bone cholesteatoma, selecting the surgical approach based on lesion extent, inflammatory status, and the need for hearing preservation is important for optimizing outcomes. In cases with extensive disease and significant inflammation, a staged strategy incorporating the middle cranial fossa approach may represent a safe and effective option for achieving disease eradication while minimizing the risk of inner ear dysfunction and postoperative complications.

## 1. Introduction

Sanna et al. classified petrous bone cholesteatomas into the following five categories: Class 1, supralabyrinthine; Class 2, infralabyrinthine; Class 3, infralabyrinthine–apical; Class 4, massive; and Class 5, apical. All types are located deep within the complex anatomy of the temporal bone, making surgical access and visualization challenging [1]. According to this classification system, the supralabyrinthine class (44%) is the commonest of all classes [2]. Supralabyrinthine petrous bone cholesteatoma is usually congenital, but it may also arise secondarily from medial extension of an acquired epitympanic (pars flaccida) cholesteatoma along the supralabyrinthine corridor. Distinguishing between these two origins is clinically important because acquired cases typically demonstrate continuity of the cholesteatoma matrix from the pars flaccida or attic to the supralabyrinthine petrous apex, whereas congenital cases usually arise as isolated medial lesions without such continuity. This lesion is centered around the geniculate ganglion and has limited medial extension beyond the otic capsule [3, 4]. Petrous bone cholesteatomas are located medial to the otic capsule, and complete removal of the lesion often requires drilling through the otic capsule. However, supralabyrinthine petrous bone cholesteatoma is characterized by a lesion situated superior and medial to the superior and lateral semicircular canals, with partial preservation of the osseous labyrinth in some cases [1]. Because of this anatomical configuration, the potential for hearing preservation is generally greater than in other subtypes of petrous bone cholesteatoma, making the selection of an appropriate surgical approach essential for achieving optimal outcomes.

The present case involved an acquired pars flaccida cholesteatoma that had extended into the supralabyrinthine region by entering the space between the superior semicircular canal and the middle cranial fossa (MCF) dura, while inner ear function remained completely preserved. The disease exhibited a highly inflammatory nature, with persistent otorrhea lasting for more than 2 years. We report this case because it was successfully managed using a two‐stage surgical strategy with a MCF approach. Considerations for selecting the most appropriate surgical approach for such cases are also discussed.

## 2. Case Presentation

A 31‐year‐old man presented with a chief complaint of left otorrhea. He had experienced persistent left‐sided otorrhea for 2 years and was referred to our department after cholesteatomatous otitis media was suspected by his primary physician. On otoscopic examination, granulation tissue and otorrhea were observed in the left pars flaccida (Figure 1A). No facial nerve dysfunction was noted (House–Brackmann Grade I), and the patient did not report any vestibular symptoms such as vertigo or imbalance. Pure‐tone audiometry revealed conductive hearing loss with an air–bone gap (ABG) of 35 dB (three‐frequency average method; Figure 1B). Air‐conduction (AC) thresholds at 0.5, 1, and 2 kHz were 30, 30, and 50 dB, respectively, while bone‐conduction (BC) thresholds at the same frequencies were 5, 5, and 30 dB, respectively. Speech audiometry was not performed. The three‐part method is an audiological technique used to quantify the ABG by averaging the differences between AC and BC thresholds at 0.5, 1, and 2 kHz. High‐resolution computed tomography (HRCT) of the temporal bone demonstrated diffuse soft‐tissue density extending from the left tympanic cavity to the mastoid air cells and further to the supralabyrinthine petrous apex (Figure 2A, B). The lesion was located superior to the superior semicircular canal and adjacent to the MCF dura, with preservation of the otic capsule. Preoperative imaging parameters included a slice thickness of 0.63 mm and axial and coronal reconstructions. Postoperative evaluation was performed using non–echo‐planar diffusion‐weighted magnetic resonance imaging (non–EPI DWI MRI) with a slice thickness of 1 mm, which is considered highly sensitive for detecting residual cholesteatoma. A staged surgical strategy was planned.

**FIGURE 1:**
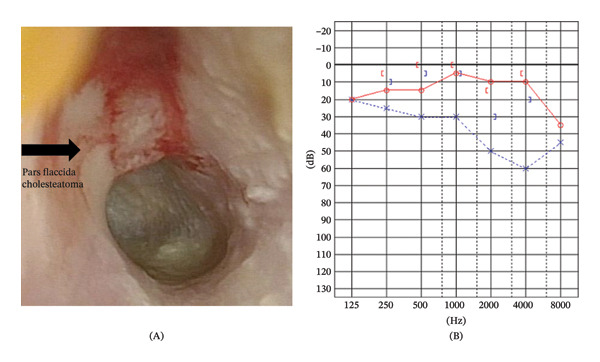
Preoperative findings. (A) Left tympanic membrane. Arrow: pars flaccida. (B) Hearing loss with an air–bone gap of 35 dB (the three‐part method).

**FIGURE 2:**
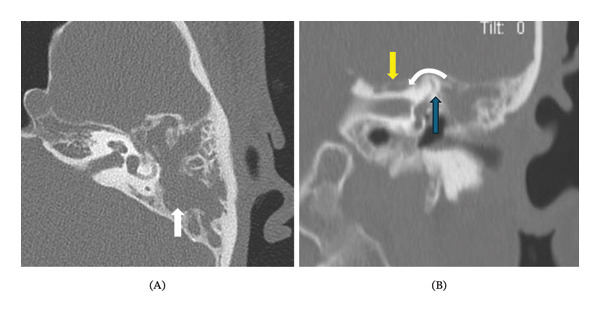
Preoperative CT findings. (A) Diffuse soft‐tissue density extending from the left tympanic cavity to the mastoid air cells. (B) White arrow: extending to the supralabyrinthine petrous apex. Yellow arrow: supralabyrinthine petrous apex. Blue arrow: superior semicircular canal.

First‐stage surgery: A canal wall–down tympanomastoidectomy was performed via a transmastoid approach. Intraoperatively, extensive inflammatory changes were observed throughout the mastoid air cells. The ossicular chain was initially intact. The incudostapedial joint was disarticulated, and the incus was removed to allow safe excision of the cholesteatoma. There was no exposure of the facial nerve. The cholesteatoma was not in contact with the geniculate ganglion. It extended medially along the plane between the superior semicircular canal and the MCF dura, spreading into the supralabyrinthine region. The cholesteatoma extending supralabyrinthine was left in situ without removal. Second‐stage surgery: A transmastoid approach confirmed the absence of recurrent disease in the tympanic cavity and mastoid. Ossicular reconstruction was performed using cortical bone, which corresponds to ossiculoplasty type “Osm” according to the standardized reporting system for outcomes after middle ear and mastoid surgery proposed by Yung et al. [5]. Subsequently, the cholesteatoma extending over the labyrinth was removed via the MCF approach. The MCF approach was performed with careful identification of anatomical landmarks. The MCF approach was performed with careful identification of anatomical landmarks. Following temporal craniotomy, the dura mater was gently elevated from the floor of the MCF, with particular care taken to avoid dural injury. The middle fossa dura was elevated in a posterior‐to‐anterior direction, extending from the level of the arcuate eminence posteriorly to the region just anterior to the greater superficial petrosal nerve (GSPN) anteriorly, and from the petrous ridge medially to the lateral edge overlying the tegmen. This extent of dural elevation provided sufficient exposure of the superior surface of the petrous bone overlying the supralabyrinthine compartment, while minimizing temporal lobe retraction. Cottonoid patties were placed to protect the dura and underlying neural structures, and meticulous hemostasis was achieved using bipolar coagulation and bone wax as needed. After identification of the GSPN and the arcuate eminence, the supralabyrinthine compartment was entered between these two landmarks, anterior to the superior semicircular canal and posterior to the geniculate ganglion. Upon dural elevation, the bone overlying the supralabyrinthine cells was found to be only partially preserved, and the majority of the cholesteatoma matrix was already exposed on the superior surface of the petrous bone. The remaining bony covering was carefully removed under continuous microscopic visualization to fully delineate the extent of the matrix within the supralabyrinthine space. The cholesteatoma matrix was then removed primarily en bloc, with piecemeal dissection performed only in limited areas where the matrix was firmly adherent to the surrounding bone. Complete removal was confirmed under direct microscopic inspection of the supralabyrinthine cavity, with particular attention paid to the blind corners of the petrous apex. Intraoperatively, the cholesteatoma was located posterior and medial to the geniculate ganglion and medial to the labyrinthine segment of the facial nerve, without direct contact with either the geniculate ganglion or the GSPN. The facial nerve and the otic capsule remained intact throughout the procedure. Special attention was paid to dural handling throughout the procedure to prevent cerebrospinal fluid leakage, and no dural tear or cerebrospinal fluid leakage was encountered intraoperatively.

### 2.1. Postoperative Course

No intraoperative or postoperative complications occurred. At 22 months postoperatively, the AC threshold improved to 21.7 dB as measured by the three‐frequency pure‐tone average (Figure 3B), and no tympanic membrane findings suggestive of recurrence were observed. At 4 months postoperatively, non–EPI DWI MRI (slice thickness, 1 mm) demonstrated no evidence of residual cholesteatoma (Figure 3C).

**FIGURE 3:**
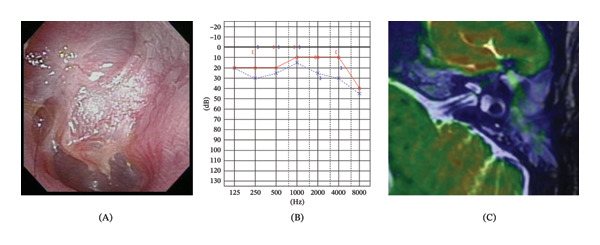
Postoperative findings. (A) Tympanic membrane. (B) Hearing loss with an air–bone gap of 21.7 dB (the three‐part method). (C) Non–EPI diffusion‐weighted MRI (1‐mm slice thickness) showed no evidence of residual cholesteatoma.

Based on the combination of clinical, radiological, and intraoperative findings, the present case was classified as an acquired pars flaccida cholesteatoma with secondary extension to the supralabyrinthine petrous apex, rather than a congenital supralabyrinthine cholesteatoma. Specifically, otoscopic examination demonstrated granulation tissue and otorrhea at the left pars flaccida, indicating a pars flaccida origin. HRCT of the temporal bone showed a continuous soft‐tissue density extending from the left tympanic cavity and epitympanum through the mastoid air cells into the supralabyrinthine petrous apex (Figure 2A, B). Intraoperatively, the cholesteatoma was confirmed to be contiguous from the attic through the mastoid and extended medially along the plane between the superior semicircular canal and the MCF dura into the supralabyrinthine region. Taken together, these findings supported the diagnosis of medial extension of an acquired pars flaccida cholesteatoma, rather than a primary congenital lesion of the petrous apex.

## 3. Discussion

The fundamental principle of surgical management of cholesteatoma is complete eradication of the disease; however, when inner ear function is preserved, hearing preservation should be pursued whenever possible. To date, the following surgical techniques have been reported for the purpose of hearing preservation in the management of supralabyrinthine petrous bone cholesteatoma. Transmastoid trans–superior semicircular canal approach: For small, well‐localized lesions, the transmastoid trans–superior semicircular canal approach is particularly advantageous. By partially removing the superior semicircular canal to access the lesion, this technique minimizes labyrinthine disruption and has been associated with favorable long‐term BC hearing outcomes [6]. Superior semicircular canal plugging using the underwater technique: When inner ear function is preserved and hearing preservation is desired, options include the translabyrinthine approach with superior semicircular canal plugging using the underwater technique. Previous reports have demonstrated the efficacy of this technique in the management of superior semicircular canal dehiscence [7, 8], suggesting that it may also be advantageous in surgical treatment of supralabyrinthine petrous bone cholesteatoma. Partial labyrinthectomy: In cases where the lesion abuts the bony labyrinth but the membranous labyrinth remains intact, partial labyrinthectomy may be employed. This advanced technique involves selective removal of the bony labyrinth while preserving the membranous structures, enabling complete disease removal with the possibility of maintaining functional hearing [9]. The MCF approach: This approach is performed through a temporal craniotomy and allows preservation of hearing by avoiding direct manipulation of the cochlea and labyrinth. Its major advantages include an excellent surgical view and the potential for hearing preservation [2].

The following considerations are made to determine which surgical approach is most appropriate based on the extent and anatomical location of the lesion among the techniques described above. Selection of the surgical approach for supralabyrinthine petrous bone cholesteatoma should be individualized according to lesion size and extent, preservation of inner ear function, the need for a broad surgical field, and the risk of inner ear injury associated with each technique. When the lesion is very small and well localized, allowing preservation of the membranous labyrinth, and inner ear function remains intact, partial labyrinthectomy may be considered. When the lesion is larger and preservation of the membranous labyrinth is not feasible, but inner ear function is still maintained, the transmastoid trans–superior semicircular canal approach or superior semicircular canal plugging using the underwater technique may allow removal of the lesion while attempting to preserve inner ear function. However, all three of these transtemporal techniques (Figure 4A) carry a substantial risk of inner ear function loss. In contrast, the MCF approach (Figure 4B) is particularly suitable for lesions with wide supralabyrinthine extension and/or lesions requiring a broad surgical field, when preservation of inner ear function is essential because it provides direct access to the supralabyrinthine compartment without traversing the labyrinth. This technique carries a low risk of inner ear function loss [10]. Therefore, rather than being indicated regardless of lesion size, the MCF approach is best regarded as the approach of choice when wide exposure of the supralabyrinthine compartment is required and inner ear function must be preserved. In the present case, the cholesteatoma extended widely into the supralabyrinthine compartment, requiring a broad surgical field, and preservation of inner ear function was essential. Therefore, the MCF approach was considered the most appropriate option. However, the approach requires careful management of temporal lobe retraction to minimize the risk of postoperative complications such as seizures, cerebrospinal fluid leakage, or facial nerve dysfunction.

**FIGURE 4:**
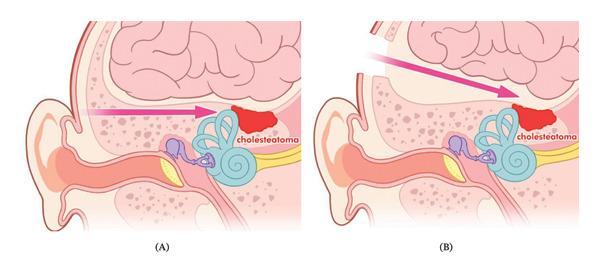
Surgical approach to the medial portion of the superior semicircular canal. (A) Transmastoid approach via the superior semicircular canal. (B) Middle cranial fossa approach.

A decision must be made regarding whether to complete the surgery in a single stage or to adopt a staged approach. The present patient had experienced persistent otorrhea for several years and demonstrated marked inflammatory activity; therefore, the degree of inflammation immediately before surgery was an essential factor in determining the treatment strategy. Given the severe inflammatory condition, the risks of residual disease, recurrence, and intracranial complications were anticipated to be high. In addition, severe inflammation was thought to obscure the true extent of the cholesteatoma matrix, making precise identification and safe removal during a single‐stage surgery technically challenging.

Accordingly, the first‐stage procedure aimed to achieve adequate control of inflammation by removing the cholesteatoma in the mastoid and tympanic cavity via a transmastoid approach. After an interval of approximately 6 months, the second‐stage surgery was performed using the MCF approach to excise the supralabyrinthine petrous bone cholesteatoma. During this second‐stage operation, the mastoid and tympanic cavity were also inspected for recurrence, and ossicular chain reconstruction was undertaken. After confirmation of adequate inflammatory control and stabilization of the operative field, definitive disease removal via the MCF approach was performed as the second stage 6 months later. This interval was judged sufficient to allow tissue recovery and to permit safer and more reliable identification of the residual supralabyrinthine matrix. This staged strategy may reduce the risk of recurrence and decrease the likelihood of complications. In cases with pronounced inflammation such as the present one, a two‐stage approach represents a reasonable and potentially advantageous treatment option.

Beyond approach selection and the staged strategy, several additional considerations contributed to minimizing the risk of residual or recurrent cholesteatoma in this anatomically challenging location. First, the first‐stage tympanomastoidectomy not only controlled the active infection but also provided a clean, well‐delineated operative field for the second‐stage MCF approach, allowing more reliable identification of the residual supralabyrinthine matrix. Second, at the second‐stage MCF approach, the cholesteatoma matrix was directly visualized under continuous microscopic magnification, and the matrix was removed primarily en bloc, with piecemeal dissection only in limited areas of firm adhesion. This strategy was intended to minimize fragmentation of the matrix and reduce the risk of residual epithelial remnants. After removal, the supralabyrinthine cavity was carefully inspected, with particular attention paid to the blind corners of the petrous apex. Third, structured postoperative surveillance was performed using non–EPI DWI MRI with a 1‐mm slice thickness, which is highly sensitive for detecting small foci of residual or recurrent cholesteatoma, in combination with serial otoscopic and audiometric follow‐up. We believe that, together with the wide exposure provided by the MCF approach and the staged surgical strategy, these considerations contributed to reducing the risk of recurrence in the present case.

Audiologically, the preoperative BC threshold was mildly elevated despite preservation of the otic capsule on HRCT. The patient had a 2‐year history of persistent otorrhea, and long‐standing middle ear inflammation is known to affect the inner ear secondarily through the round window membrane, leading to mild high‐frequency sensorineural hearing loss even in the absence of overt labyrinthine erosion [10].

### 3.1. Limitations

This report describes a single case; therefore, its external validity is limited. The clinical outcome observed here cannot be generalized to all cases of supralabyrinthine petrous bone cholesteatoma. In addition, comparative efficacy and safety among different surgical approaches cannot be inferred from a single‐case experience. Accordingly, this report should be regarded as educational and hypothesis‐generating rather than providing definitive conclusions. A 4‐month postoperative MRI and tympanic membrane findings at 22 months postoperatively are insufficient to confirm long‐term disease eradication. Longer follow‐up is required to adequately assess recurrence.

## 4. Conclusion

In supralabyrinthine petrous bone cholesteatoma, careful selection of the surgical approach based on lesion extent, inflammatory status, and the necessity of hearing preservation is essential for optimizing outcomes. In cases with extensive disease and significant inflammation, a staged surgical strategy incorporating the MCF approach may provide a safe and effective means of achieving complete disease eradication while minimizing the risk of inner ear dysfunction and postoperative complications.

## Author Contributions

Hisashi Sugimoto: conceptualization, methodology, investigation, surgery, and writing–original draft.

Manabu Inaba: supervision and writing–review and editing.

Goki Nagasaki: data curation and investigation.

Tomokazu Yoshizaki: supervision and project administration.

## Funding

This research did not receive any specific grant from funding agencies.

## Ethics Statement

This study was conducted in accordance with the Declaration of Helsinki. The Institutional Review Board of Kanazawa University determined that ethical approval was not required for this single case report. Written informed consent for publication, including clinical data and images, was obtained from the patient.

## Conflicts of Interest

The authors declare no conflicts of interest.

## Data Availability

The data that support the findings of this study are openly available in KURA at https://kanazawa-u.repo.nii.ac.jp/.
